# Supplementary material for: Unraveling the temperature dynamics and hot electron generation in tunable gap-plasmon metasurface absorbers
Source: Nanophotonics. 2022 Apr 12;11(17):4037–52. doi: 10.1515/nanoph-2022-0048 (PMC11501958; doi:10.1515/nanoph-2022-0048)
Supplement: Supplementary file 1 — Supplementary Material [file j_nanoph-2022-0048_suppl.pdf]

Supplementary Material

Unraveling the temperature dynamics and hot electron generation in tunable gap-plasmon metasurface absorbers

*Larousse Khosravi Khorashad and Christos Argyropoulos**

Department of Electrical and Computer Engineering, University of Nebraska-Lincoln, Lincoln, NE, 68588, USA
*E-mail: christos.argyropoulos@unl.edu

Numerical Modeling Method Details

In this work, we used COMSOL Multiphysics for all the performed numerical calculations. COMSOL Multiphysics is a commercial software based on the finite element method. The Radio Frequency (RF) module was used to calculate the plasmonic resonances along with the electromagnetic heating computed by Equation 6 and generation rate of high energy (hot) electrons given by Equation 8 in the main text. The entire geometry was excited with an electromagnetic wave via a port excitation propagating from the top to the bottom. Periodic boundary conditions were placed on both geometry sides located at a distance equal to the structure's periodicity. Furthermore, we used two separate Heat Transfer (HT) COMSOL modules to solve the TTM coupled partial differential equations (Equations 1 and 2 in main text) and, subsequently, calculate the electron and lattice temperatures in time domain. Note that Equations 1 and 2 do not have the conventional heat equation form. The additional terms, such as the electron-lattice coupling factor and the source term in Equation 1, are introduced as supplementary heat sources in the first HT module dedicated to solving the electron temperature. Similarly, the coupling factor in Equation 2 is introduced as a heat source in the second HT module, which is used to compute the lattice temperature. The two HT modules are coupled by the electron and lattice temperature variables. The computed electromagnetic heating derived by Equation 6 using the RF module was coupled to the HT module via the heat source definition.

Periodic conditions were chosen for the metallic parts and the entire surrounding was kept at room temperature as the initial temperature condition. The heat source was applied to the metallic parts since only them possess an imaginary part of permittivity. All parts of the geometry in RF and HT modules are coupled via the Multiphysics Electromagnetic Heating (emh) module.

The electric current density $\vec{j}(\vec{r})$ in the nonlocal model of Equation 7 is computed by using the weak form partial differential equation COMSOL module. Then, the calculated current density $\vec{j}(\vec{r})$ is introduced as a weak contribution to the COMSOL frequency domain electromagnetic solver. This weak contribution is added as an additional polarization term in the electromagnetic wave equation that now becomes: $\nabla \times \nabla \times \vec{E}(\vec{r}) - k_0^2\vec{E}(\vec{r}) = i\omega\mu_0\vec{j}(\vec{r})$, which is solved by the COMSOL frequency domain electromagnetic solver. Finally, the continuity in the normal component of the electric displacement field between silver and the dielectric nanogap needs to be manually introduced in the COMSOL weak form module as an additional Dirichlet boundary condition. This is due to the presence of spatial derivatives in the nonlocal current density equation given by Equation 7.

Figures and Tables

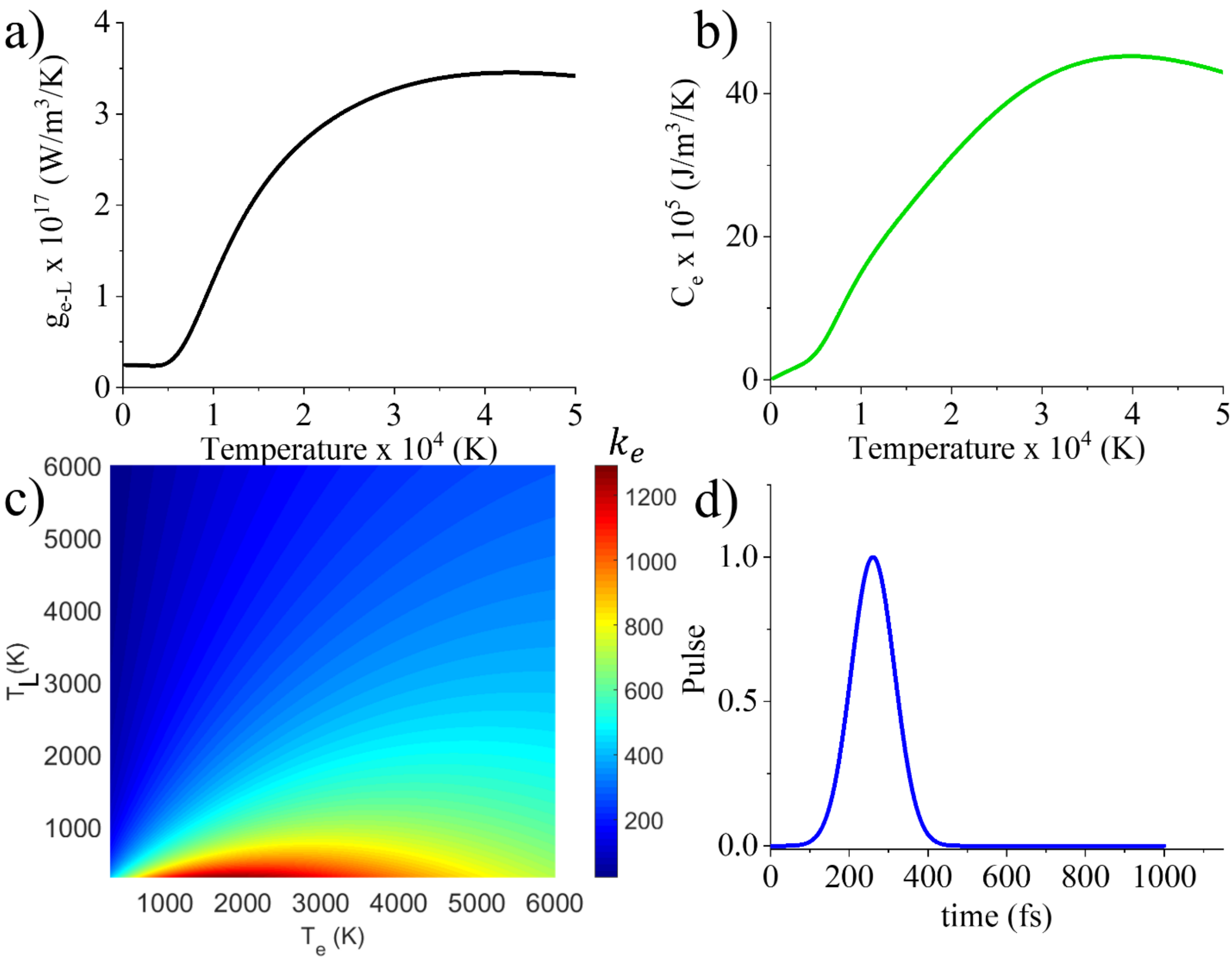

Figure S1. a) Electron-lattice coupling factor, b) electron heat capacity, and c) electron thermal conductivity derived from Equation (3). The material characterized by these parameters is silver. d) The envelope of the femtosecond Gaussian pulse with maximum amplitude at $t = 3\tau$, where $\tau = 130fs$.

Table S1. Various parameters used in our simulations.

Parameter	Value	Unit
Density (ρ) of silver	10.49	g/m^3
Density (ρ) of alumina	3.95	g/m^3
Density (ρ) of air	1205	g/m^3
Lattice thermal conductivity (k_L) of silver	428[W/(m*K)]	W/m/K
Thermal conductivity (k) of alumina	18[W/(m*K)]	W/m/K
Thermal conductivity (k) of air	0.024	W/m/K
Specific heat capacity (C_p) of silver (lattice)	0.233	J/kg/K
Specific heat capacity (C_p) of alumina	880	J/kg/K
Specific heat capacity (C_p) of air	1005	J/kg/K
Permittivity of silver	Ref.[1]	-
Permittivity of alumina	3.13	-
Permittivity of air	1	-

Table S2. Silver parameters used in Equation 3, 9 and 10 taken from Refs.[2,3]

Parameter	Value	Unit
ω_p	9.01	eV
f_0	0.845	-
A	0.932×10^7	1/s/K^2
B	1.02×10^{11}	1/s/K

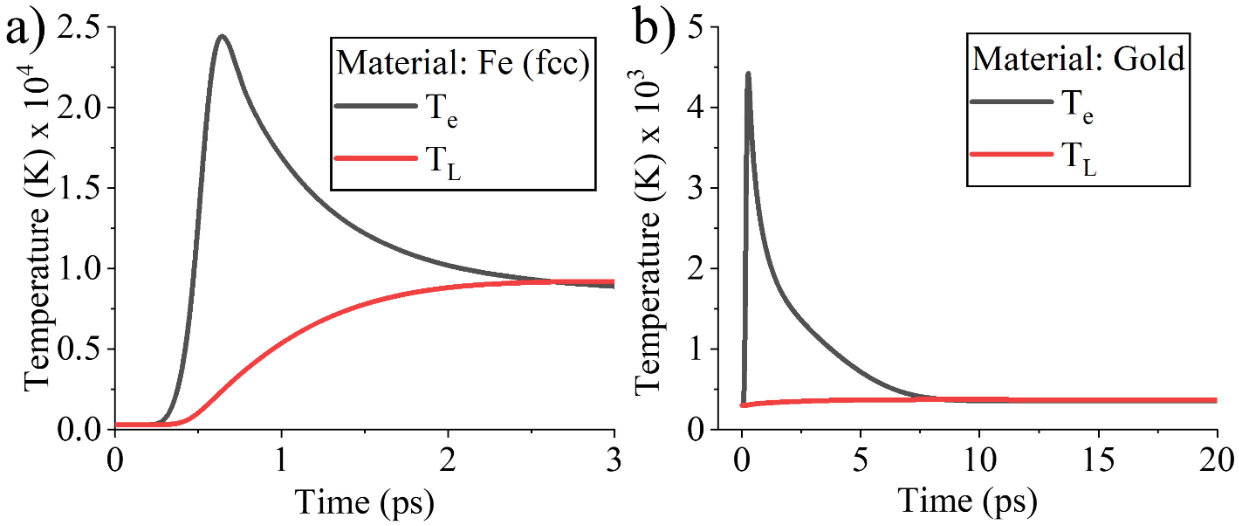

Figure S2. Electron and lattice temperatures computed by the current multiphysics simulations and obtained at the surface of a) bulk iron (fcc) and b) 200nm thick flat gold film. The results perfectly agree with previous published works based on analytical approaches.[2,4] All the parameters used in these simulations are identical to the previous analytical works.[2,4]

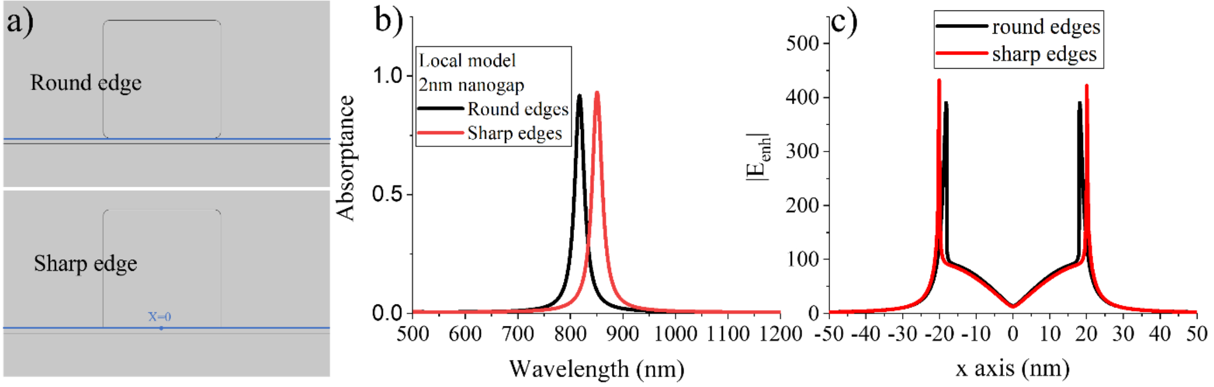

Figure S3: a) Round (top panel) and sharp (bottom panel) edge nanostripe geometries in the case of a 2nm nanogap metasurface absorber. The round scenario is the same with the one used in the main paper. b) Absorptance spectrum of the two geometries demonstrated in (a). c) Electric field enhancement computed along the blue line in (a) (bottom of the nanostripe) at each corresponding absorptance resonance: 818nm (round edge) and 852nm (sharp edge).

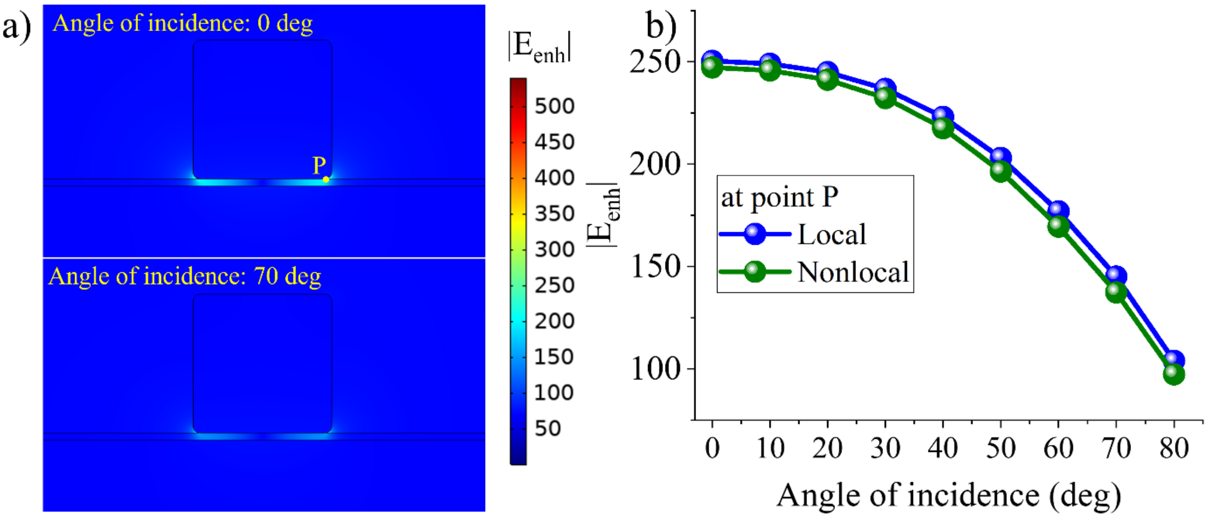

Figure S4: a) Computed electric field enhancement distribution by using the local model for normal (top panel) and 70° oblique (bottom panel) incident illumination. b) Electric field enhancement as a function of incidence angle for local and nonlocal models computed at point P shown in caption (a). These calculations are for the 2nm nanogap metasurface.

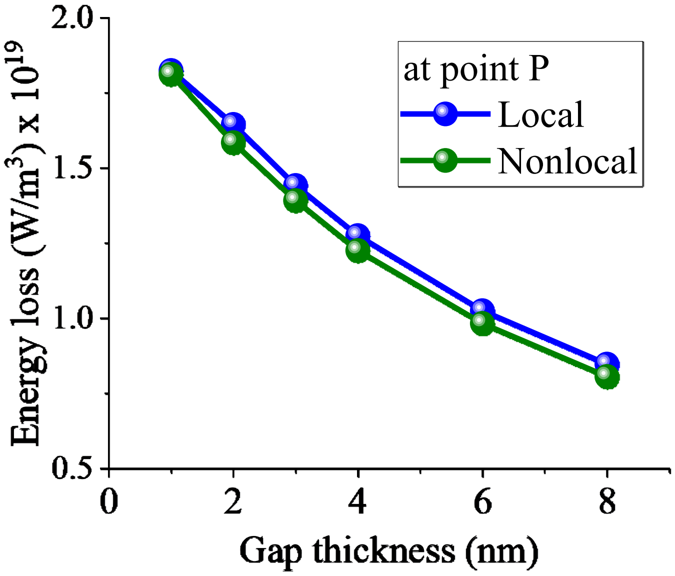

Figure S5: Average energy loss delivered to the metallic components of the nanostructure computed at point P shown in Figure 3(c) for a 2nm nanogap metasurface illuminated by a laser intensity of 3GW/cm^2 in the case of local and nonlocal models.

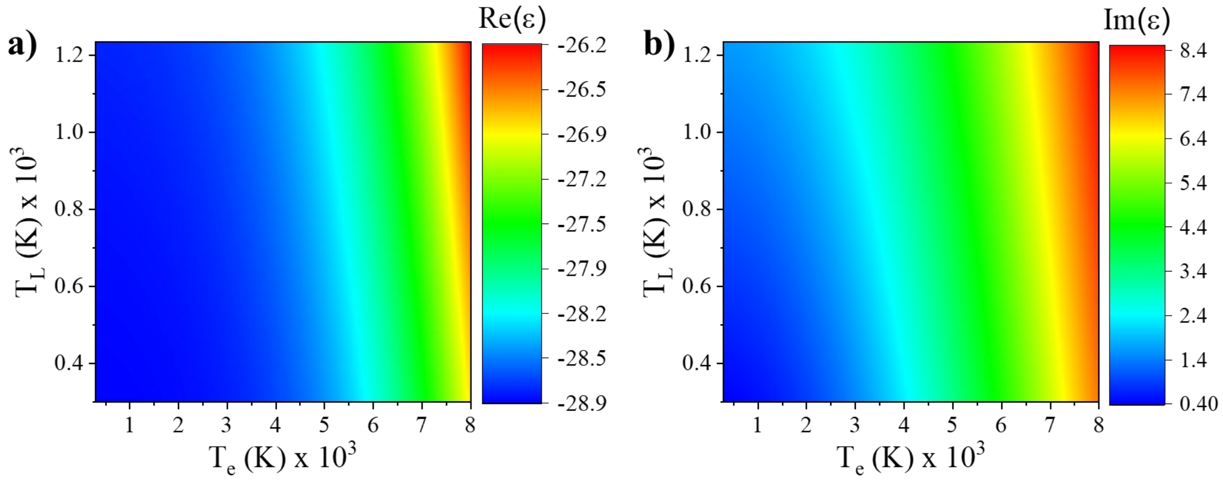

Figure S6: (a) Real and (b) imaginary parts of silver permittivity plotted as a function of electron (x-axis) and lattice (y-axis) temperatures. The results are computed under femtosecond laser illumination at the resonance wavelength (818nm) of a 2nm nanogap metasurface.

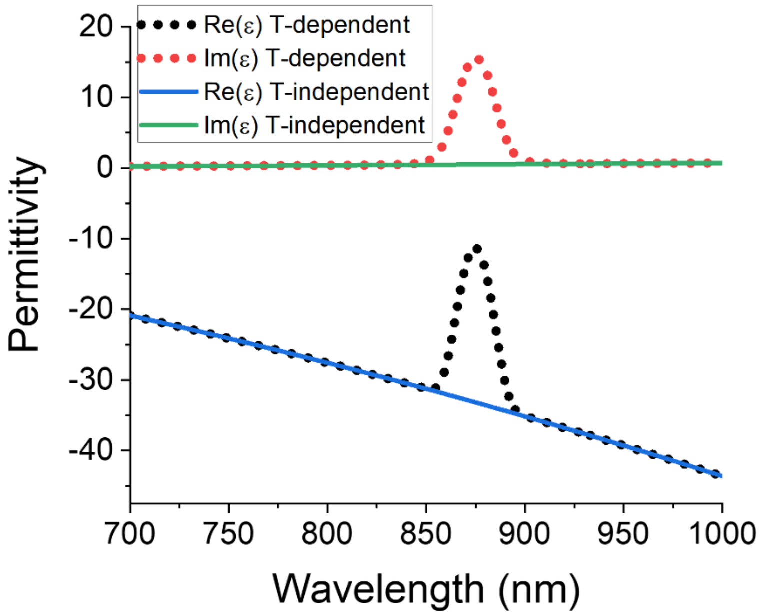

Figure S7. The local temperature dependent real and imaginary parts (dashed lines) of silver relative permittivity at point P (see inset of Figure 2(b)) derived from the TTM under femtosecond laser illumination ($I_0 = 5GW/cm^2$) combined with Equations 9 and 10 in the main paper for a $2nm$ thick gap metasurface design. The silver permittivity at room temperature is also depicted with the solid lines. The electron temperature reaches its maximum value at the resonance of this nanostructure ($\sim 880\ nm$), leading to a considerable increase in the damping factor computed by Equation 10 in the main paper. This effect results in significant changes in the silver's complex permittivity values around the resonance wavelength, as demonstrated in Figure S7, while the room temperature (temperature-independent) permittivity remains unaltered.

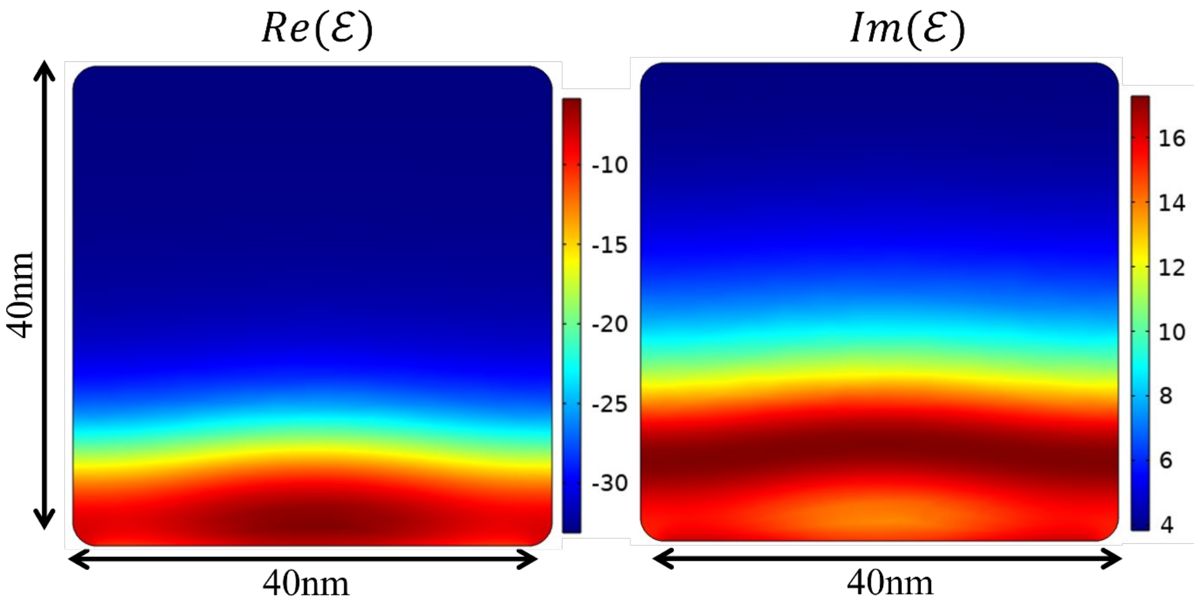

Figure S8. Spatially varying real and imaginary parts of silver permittivity plotted in a cross section of the silver nanostripe under femtosecond laser illumination ($I_0 = 5\ GW/cm^2$). These permittivity values are obtained at the resonance wavelength of $880nm$ and exactly at the Gaussian pulse peak. The silver substrate does not have a pronounced permittivity change (not shown here) because is mainly used as the reflector in the formed nanocavity and has much larger dimensions compared to the nanostripe.

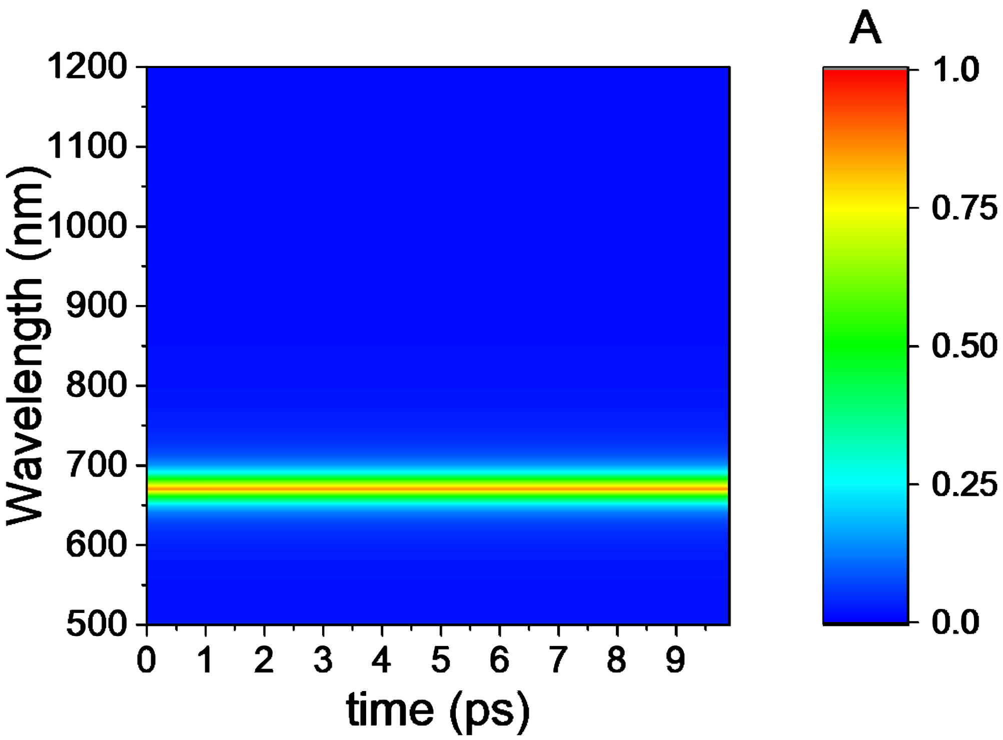

Figure S9. Computed absorptance distribution versus time and wavelength obtained by using the temperature varying silver permittivity values defined by Equation 9 in the main paper for 4nm gap thickness plasmonic absorber. Femtosecond ultrafast laser illumination is used with laser input intensity equal to $I_0 = 5\ GW/cm^2$. No temporal change in the absorption spectrum is obtained on vast contrast to Figure 7 in the main paper (2nm gap plasmonic absorber case).

References

[1] P.B. Johnson, R.W. Christy, Optical Constants of the Noble Metals, Phys. Rev. B. 6 (1972) 4370–4379.

[2] A.M. Chen, H.F. Xu, Y.F. Jiang, et al., Modeling of femtosecond laser damage threshold on the two-layer metal films, Applied Surface Science. 257 (2010) 1678–1683.

[3] A.D. Rakić, A.B. Djurišić, J.M. Elazar, M.L. Majewski, Optical properties of metallic films for vertical-cavity optoelectronic devices, Appl. Opt., AO. 37 (1998) 5271–5283.

[4] G.D. Tsibidis, A. Mimidis, E. Skoulas, et al., Modelling periodic structure formation on 100Cr6 steel after irradiation with femtosecond-pulsed laser beams, Appl. Phys. A. 124 (2017) 27.
